# Epstein-Barr Virus-Positive Primary Central Nervous System Lymphoma in Adult-Onset Still’s Disease: A Case Report

**DOI:** 10.31138/mjr.290424.ens

**Published:** 2024-12-31

**Authors:** Tomohiro Yoshida, Keisuke Nishimura, Yoko Akaike, Hiroyuki Murabe

**Affiliations:** 1 Department of Endocrinology and Rheumatology, Kurashiki Central Hospital, Okayama, Japan,; 2 Graduate School of Health Management, Keio University, Kanagawa, Japan,; 3 Department of Rheumatology and Clinical Immunology, Kobe University Graduate School of Medicine, Kobe, Japan,; 4 Department of Anatomic Pathology, Kurashiki Central Hospital, Okayama, Japan

**Keywords:** adult-onset Still’s disease, immunosuppressed state, ferritin, diffuse large B-cell lymphoma, central nervous system

**Figure 1.**
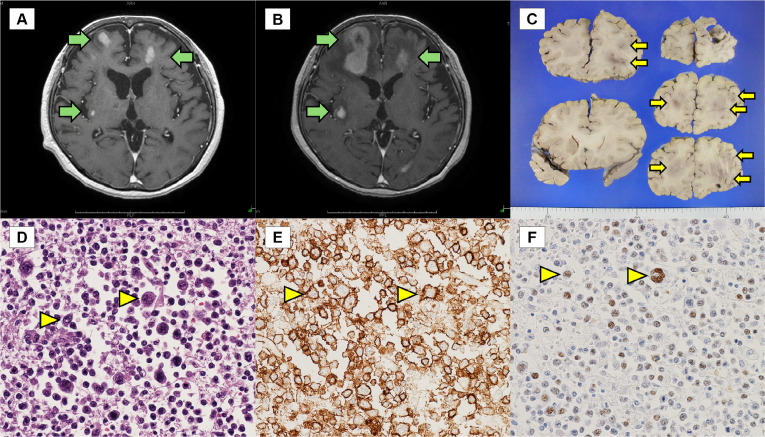
MRI findings of the brain on post-contrast T1-weighted axial images (**A, B**) and findings from an autopsy (**C–F**). **(A)** MRI findings when the patient had left upper extremity paralysis. Multiple shadows show uniform contrast effects (green arrow). **(B)** MRI findings obtained 1 month after the patient had left upper extremity paralysis. The known shadows have grown in a short time (green arrow). **(C)** Macroscopic findings of the brain. Several centimetre-sized friable masses with indistinct borders are present in the cerebrum (yellow arrow). **(D)** Autopsy findings of brain parenchyma. Haematoxylin and eosin staining of the cerebrum (magnification 400×). A mixed proliferation of medium and large lymphoid cells can be noted (yellow arrowhead). **(E)** Autopsy findings of brain parenchyma. Immunohistochemical staining for CD20 (magnification 400×). Neoplastic cells appear positive for CD20 (yellow arrowhead). **(F)** Autopsy findings of brain parenchyma. In situ hybridisation for EBV-encoded small RNA (magnification 400×). Neoplastic cells show nuclear labelling (yellow arrowhead). MRI: magnetic resonance imaging; EBV: Epstein-Barr virus.

A 77-year-old woman presented with a 3-month history of spiked fever, arthralgia, and erythema. Her medical history was insignificant. Blood tests revealed C-reactive protein, 20.35 mg/dL; ferritin, 13,816 ng/mL; and white blood cell, 32,100/μL (neutrophils: 94%). Autoantibody screening, infectious disease tests including human immunodeficiency virus, imaging tests of the head and trunk, bone marrow examination, and skin biopsy revealed no specific findings. She was diagnosed with adult-onset Still’s disease (AOSD). High-dose prednisolone, methotrexate, cyclosporine, and tocilizumab improved her clinical state. Ferritin levels decreased to 2,279 ng/mL. We judged that she had achieved remission and tapered prednisolone. Two months later, hyperferritinaemia (10,525 ng/mL), fever, and elevated inflammatory response recurred. We adjusted her medication considering AOSD relapse; however, her clinical state remained unimproved. Subsequently, she experienced left upper extremity paralysis, facial and limb convulsions, and confusion. Magnetic resonance imaging revealed multiple shadows with uniform contrast effects, primarily in the pons, cerebellum, and cerebral white matter (**Figure 1A**), with low and high signals on T1- and T2-weighted images, respectively. The shadows grew rapidly (**Figure 1B**) and she died 160 days after admission. An autopsy revealed that medium and large lymphoid cells (CD20- and EBER-positive) had infiltrated the cerebrum, cerebellum, and pons (**Figure 1C–F**). No lymphoma involvement was found in other organs. The final diagnosis was Epstein–Barr virus-positive diffuse large B-cell lymphoma of the central nervous system.

Malignancy during AOSD treatment has many possible causes.^1^ We hypothesised that immunosuppression was a cause in our case since Epstein–Barr virus reactivation was indicated. Abscesses are commonly considered when brain lesions appear in immunosuppressed patients; however, immunosuppression-related malignancy should also be considered.

We concluded that her clinical state (including recurrent hyperferritinaemia), considered AOSD relapse, represented initial symptoms of lymphoma. This case highlights the importance of broadening differential diagnosis when AOSD becomes refractory to treatment.
